# Functional Orthodontic Therapy for Mandibular Condyle Fracture: A Systematic Review

**DOI:** 10.3390/medicina60081336

**Published:** 2024-08-16

**Authors:** Christoph-Ludwig Hennig, Franziska Krause, Ann Nitzsche, Konrad Tolksdorf, Markus Reise, Stefan Kranz, Marco Dederichs, Stefan Schultze-Mosgau, Collin Jacobs

**Affiliations:** 1 Department of Orthodontics, Center of Dental Medicine, University Hospital Jena, An der alten Post 4, 07743 Jena, Germany; 2 Department of Oral and Maxillofacial Surgery/Plastic Surgery, University Hospital Jena, Am Klinikum 1, 07747 Jena, Germany; 3 Department of Conservative Dentistry and Periodontics, Center of Dental Medicine, University Hospital Jena, An der alten Post 4, 07743 Jena, Germany; 4 Department of Prosthodontics, Center of Dental Medicine, University Hospital Jena, An der alten Post 4, 07743 Jena, Germany

**Keywords:** mandibular condyle fracture, functional orthopedic therapy, conservative fracture therapy, activator

## Abstract

The objective of this study was to compile the currently available evidence regarding the functional and morphologic outcomes of functional orthodontic therapy for mandibular condyle fracture. We performed searches in PubMed and Google Scholar as well as manually (IOK issues 2008–2019) using the keywords “trauma”, “TMJ”, “activator”, “condylar fracture”, “fracture”, “mandibular condylar fracture”, “occlusal splint” and “functional appliance”. Screening and analysis of study eligibility were performed using the Preferred Reporting Items for Systematic Reviews and Meta-Analyses (PRISMA). The search strategy identified 198 studies published between 1971 and 2018, with 93 studies remaining after removing duplicate hits. Of the 93 studies, 19 were included in this study, considering the inclusion criteria. There were 12 follow-up, 4 prospective, and 3 purely retrospective studies. Some of the studies showed good functional results of mandibular condyle fracture treatment, in addition to subjective patient satisfaction. The incisal edge difference could be increased to physiological ranges of >35 mm by means of activator therapy. Partial mandibular deviations to the fractured side remained post-therapy, especially after unilateral fractures. Fractures without significant dislocation and luxation showed radiographic changes in shape, described as hypoplastic and ellipsoid, in addition to good morphologic results. One study found that collum length shortened twofold after a luxated fracture compared with fractures without significant dislocation, despite activator therapy. Straightening of the fragment occurred only in the low-dislocated fragments. Overall, children showed a higher remodeling potential than adult patients. Several studies observed an improved clinical outcome for functional therapy after mandibular condyle fracture. The outcome is essentially determined by fracture type, fracture height, and age. Further studies, especially prospective studies, are necessary to improve the evidence of functional orthodontic therapy for mandibular condyle fractures.

## 1. Introduction

Mandibular condyle fracture is a fracture in the temporo-madibular area and is common in skull trauma. Overall, 72% of road traffic accidents involve head trauma, with between 70 and 80% of skull fractures occurring in the lower jaw [1]. Mandibular fractures occur in the joint process and collum region in 30–50% of cases [2,3,4]. Of these, about 80% of mandibular condyle fractures are unilateral, and 20% are bilateral. The aim of treating mandibular condyle fractures or other joint fractures is to restore joint function through physiological occlusion and restitution of the bony and discoligamentous structures.

Functional limitations after temporal mandibular injuries and newly occurring functional disorders as well as their optimal treatment options have repeatedly stimulated the discussion of surgical and conservative treatment alternatives. For better functional results and to avoid surgical side effects such as wound healing disorders and bleeding, conservative functional orthodontic therapy with positive development in the function and shape of the jaw joints was established [5,6,7]. Reichenbach introduced jaw fracture activators in 1934 by describing the tissue remodeling processes caused by the hypomochlion effect and the intermittent stimulus of functional masticatory forces. These therapeutic measures prevent movement restriction of the lateral pterygoid muscle. Various reports in the literature confirm this purely conservative orthodontic treatment concept, as good functional results have been achieved regardless of the fracture type [8,9,10,11,12].

Overall, the purpose of this study was to determine the currently available evidence on the functional and morphologic outcomes of functional orthodontic therapy for mandibular condyle fractures.

## 2. Materials and Methods

**Figure 1:**
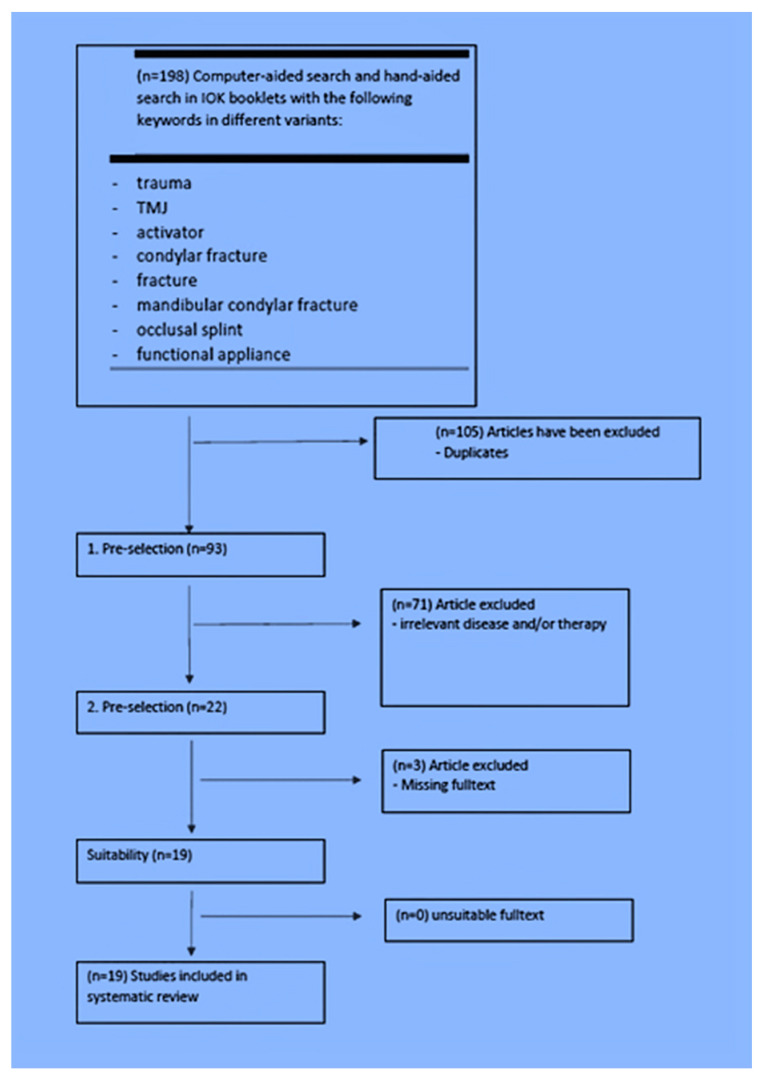
Systematic electronic search and selection of articles for review.

**Figure 2:**
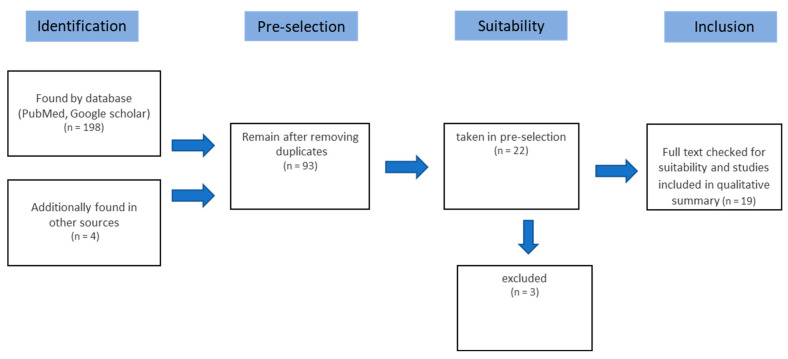
Flowchart for the different phases of the systematic review (PRISMA).

For the structured literature analysis, an electronic search was conducted in PubMed, and Google Scholar, as well as a manual search (IOK issues) using the keywords “trauma”, “TMJ”, “activator”, “condylar fracture”, “fracture”, “mandibular condylar fracture”, “occlusal splint” and “functional appliance”. The inclusion criteria for the articles included in the study were that patients with a mandibular condyle fractures were treated with functional orthodontics and the success of the treatment was reported. Furthermore, the key words mentioned had to have been mentioned in the literature. In general, only articles from 1971 up to 2018 were included in this study. A total of 198 studies on functional orthodontic treatment of mandibular condyle fractures were found. Exclusion criteria after reviewing the 198 articles included duplicates, studies with non-applicable diseases and therapies, a missing full text, and non-English literature. Screening and analysis of study admissibility were performed, considering PRISMA (Preferred Reporting Items for Systematic Reviews and Meta-Analyses) for systemic reviews (Figure 1 and Figure 2).

The literature was then reviewed and checked for relevance. The relevant articles were read and summarized in the present review.

## 3. Results

### 3.1. Selection of Studies

The search strategy identified 198 studies in the 1971–2018 period. After removing duplicates, 93 studies remained. Of the 93 studies, 19 were included in the study, considering the inclusion criteria (Figure 2). The studies included 13 follow-up studies, 4 prospective studies, and two purely retrospective studies.

### 3.2. Results of Various Studies

All studies evaluated activator therapy. Ten studies also evaluated bignatal fixation/intermaxillary fixation and three studies analyzed physiotherapy/exercise therapy for functional outcome. 

Sixteen studies included only children as patients. Three studies analyzed activator therapy for mandibular condyle fractures in adult patients. The success of therapy was verified by clinical and X-ray examination in 18 articles. In one article (Pape et al. 1973), only the clinical examination was used [13]. 

Subjectively, all studies reported a good functional outcome (Table 1). The success criteria of the studies were a mouth opening in a physiological range of >35 mm, unrestricted pro- and laterotrusion, and pain relief for the patients [12,13,14,15,16,17,18,19,20,21,22,23,24,25,26,27]. As common complications, post-therapeutic partial mandible deviations to the fractured side occurred, especially after unilateral fractures. Furthermore, morphological changes, malocclusion, and partial dysfunctions were frequently observed [12,13,14,15,16,17,18,19,20,21,22,23,24,25,26,27]. Fractures without significant dislocation and a non-significant dislocation not only showed radiologically good morphological results but also hypoplastically and ellipsoidally described shape changes. One study found that after a luxated fracture, the collum length was twice as shorten despite the administration of activator therapy compared to fractures without significant dislocation [28]. An erection of the fragment occurred only with small dislocated fragments. It is important to distinguish how subjective patient satisfaction differs from radiographic diagnosis. Patient satisfaction goes hand in hand with function. Overall, children showed a higher remodeling potential than adult patients. 

Studies that resulted in functional and/or X-ray improvement following functional orthopedic treatment of mandibular condyle fractures included the following.

Keutken et al. (1983) showed that although the functional limitations were often subjectively satisfactory, the X-ray was clearly visible. In the X-ray examination, 9 out of 28 patients (32.1%) with one-sided fractures showed ad integrum restitutio, whereas only 1 out of 15 subjects (6.7%) with double-sided fractures showed such an outcome [14]. Dislocated fractures showed greater anatomical changes in the mandibular joint, head, and neck. None of these fracture types achieved an X-ray restitutio ad integrum. Good X-ray results were found only in fractures with a low degree of dislocation. Keutken et al. (1983) supported the continuation of an anatomical–functional therapy according to the mandibular condyle fracture, since the examination showed a large absence of complaints and only minor functional dysfunctions despite anatomical changes [14]. They recommended that luxation fractures, especially double-sided fractures, be treated surgically, especially in adults. The authors also emphasized the extraordinary remodeling potential of the mandibular joint in childhood. In contrast to a child’s fracture, there is no new formation of the mandibular joint head in adults [14]. Hence, this age group should continue to be studied separately. In the X-ray examination, there were clear morphological differences depending on the treatment, but these differences were not relevant in terms of function and subjective well-being. 

Schendel et al. (1991) found that previously immobilized patients were more frequently found to have morphologically and functionally unsatisfactory results [15]. However, this patient population was generally older and described as less cooperative.

Wichelhaus et al. (1998) studied 14 patients with dislocated mandibular condyle fractures treated with bignath fixation and spring activator [16]. The central object of the study was the assessment of mouth opening under therapy. Therapy with a spring activator resulted in good to very good functional recovery in all cases. The cutting-edge difference and mouth opening were significantly increased, with the greatest effect observed in the first 4 weeks of treatment. The effect of functional recovery is described as good in adolescents. In adults, the prognosis is slightly less favorable. However, these patients also had severe facial and muscular injuries and multiple injuries in the sense of polytrauma [16].

A study by Kahl-Nieke and Fischbach (1998) investigated the relationship between patient age, fracture type, and remodeling potential of the condyle, which was previously demonstrated [17]. In this study, the type of fracture had a decisive influence on the prognosis of remodeling. Shortening of the collum and excessive bone apposition were found, especially in luxated and/or deep fractures. The more severe the fracture, the more frequent the asymmetrical morphological findings. This effect was attributed to an altered muscle structure and altered muscle volume. Younger patients showed a higher remodeling potential than older patients [18]. Strobl et al. (1999) confirmed the efficacy of functional orthopedic activator treatment in 55 children with mandibular condyle fractures. During the 48-week follow-up period, no patient showed occlusal or functional impairment or pain. The remodeling of the collum was completed during the same follow-up period. No further remodeling was observed during the growth period. The functional treatment prevented late complications, such as ankylosis, facial growth, or malfunction of the jaw joint. The study showed that secondary condyle remodeling continued with a functionally adequate condyle despite a significant displacement of the condyle, followed by subsequent resorption of the proximal fragment. Complete regeneration was found in the age group of 2–6 years, while children aged 7–10 years showed incomplete regeneration—two with a moderate condyle deformity, two with reduced neck height, and 4 cases with a hypertrophic condyle deformity [19].

Choi et al. (2005) also examined children. None of the 11 subjects reported functional complaints. Two unilateral (25%) and one bilateral fracture showed slight facial asymmetries. One patient complained of joint cracking, and two patients complained of tooth misalignment. The mobility of the mandible, including mouth opening, protrusion, and laterotrusion, was unrestricted. In the computer tomography examination of the unilateral fractures, significant changes in reconstruction were evident—six (54.5%) showed incomplete remodeling, and three subjects (27.3%) showed facial asymmetries. The condyles varied from ellipsoid to concave-convex and ovate. Overall, the authors evaluated the active gate as a satisfactory treatment method, despite the radiographic condylar malposition or lack of condylar conversion processes reported by other authors [20].

Also, Wu et al. (2012) compared unilateral and bilateral fractures with regard to clinical and X-ray outcomes after functional orthopedic treatment. The patients were given a four-week bignath fixation beforehand. Both the clinical and X-ray results were satisfactory. Through therapy, the mouth opening was brought significantly into the normal range. Outstanding clinical findings were pain (n = 2), joint cracking (n = 1), and mandibular deviation (n = 2). X-rays showed complete recovery in 50% of unilateral fractures and 43% of bilateral fractures [21].

Boffano et al. (2012) investigated 14 children whose dislocated mandibular condyle fractures had been treated with intermaxillary fixation (IMF) and activators. The clinical–functional results were satisfactory in all children. Only one patient had a slight facial asymmetry remaining. In another patient, no joint cracking, pain, or subjective restrictions were reported. Radiologically, 13 X-rays showed complete recovery. The remodeling was incomplete in only one patient [22].

Zhao et al. (2014) investigated 14 children with mandibular condyle fractures in a follow-up study after functional orthopedic treatment combined with functional exercises. Here, again, all patients showed a good functional result: no restriction of mouth opening and no pain. Only one boy showed post-interventional joint cracking. Lower jaw deflection was found in two patients with unilateral fractures. All children with bilateral fractures showed complete remodeling without obvious condyle deformities. Children with unilateral fractures tended to have incomplete remodeled condyles with short and flattened condylar heads and flattened glenoidal fossa. Remodeling processes were more dependent on fracture patterns than on age. Young people also have good remodeling potential [23].

Liu et al. (2014) interviewed 30 children who were treated with splints after mandibular condyle fractures. In all cases, positive results were obtained (excellent n = 20, good n = 10). In all unilateral fractures, there was a lateral deviation to the fractured side at the maxillary mouth opening. X-rays (23 condyles) showed 19 children (19 condyles) with complete remodeling and 11 children (14 condyles) with incomplete remodeling [24]. The individual studies are summarized in Table 1.

In conclusion, a correlation between patient age, fracture type, and the remodeling potential of the condyle was demonstrated in almost all studies. Children up to six years of age showed complete regeneration, whereas older children and adolescents showed incomplete remodeling with moderate condyle deformities. However, according to the studies, the type of fracture has a decisive influence on the prognosis of remodeling. Table 2 provides an overview of the treatment options in the various studies according to fracture type.

## 4. Discussion

The 19 studies reviewed here all reported conservative treatment of mandibular condyle fractures. It should be noted that one-sided and two-sided mandibular condyle fractures were investigated, and different conservative therapies were employed via bignath fixation and/or therapy using an activator. Further, patients of different age groups with conservative therapy for mandibular condyle fractures were treated in the literature, which makes comparison even more difficult. None of the studies reported completely consistent therapy variants, therapy times, and correspondingly the same approaches to follow-up. Different parameters were used to assess the conservative therapeutic success of mandibular condyle fractures, which were related to the patient’s self-assessment and to measurable parameters. The number of patients also varied greatly. Overall, there were three different types of study design: follow-up, prospective, and retrospective studies. These different approaches made direct comparability difficult. In general, however, conservative treatment of mandibular condyle fractures achieved good results in function and patient satisfaction in all studies. Better remodeling of the jaw joints and production of function have been achieved in many studies by activator therapy.

The functional therapy approach is possible due to the enormous musculoperiosteal remodeling capacity of the condyle, especially in childhood, as has been proven in many studies [15,19,20,21,25]. This eliminates the risk of general anesthesia and complications from the surgical procedure, such as facial scarring and nerve damage. Further, hospitalization is not necessary for functional therapy. The treatment method is more patient-friendly [25] and shows good results [13,14,19,20,21,22,23,24,27,30,31].

The remodeling potential of infantile bone has been consistently postulated [15,19,20,21,25]. In the age group between two and six years, complete remodeling of the mandibular process fracture occurs, whereas in the age group between seven and twelve years, incomplete regeneration with a moderate condylar deformity, reduced collum neck height, and hypertrophic condyles is more common [19,24]. Therefore, some authors call for separate assessments of children and adults [14]. Regarding our research subjects, studies with children clearly predominate [15,19,20,21,22,23,24]. Children have optimal conditions for functional orthodontic therapy due to their high potential for musculoperiosteal remodeling. It appears that the result of the skeletal effect is generally greater in children, as the bone remodeling is supported by the growth still present. A dental effect on the teeth is not desired therapeutically, but could occur as a side effect of the activator therapy in the context of an open bite, which closes spontaneously again later as soon as the activator is ground in therapeutically or is discontinued. Functional orthodontic treatment using an activator, bionator, or spring activator is considered a recognized therapy method with good functional effects [13,15,19,20,23,27,28,31]. Zhao et al. (2014) postulated a good remodeling potential, even in adolescents. Adults are more likely to undergo surgical therapy due to their lower functional remodeling capacity [23].

In summary, the literature describes that functional orthodontic therapy can achieve a good functional outcome after TMJ process fracture [13,14,15,16,19,20,21,22,23,24,27,29,30,31]. This is also confirmed by two comparable studies which were not included in this study due to the inclusion criteria but present similar radiographic and functional treatment results [32,33]. A few patients showed post-interventional complaints. However, if complaints occurred, the most common were pain, mandibular jaw deviation at mouth opening [13,21,23,24,25], facial asymmetry [20,22], and/or temporomandibular joint clicking [20,21,22,23,25,29].

This study is limited in the direct comparability of the studies due to the large differences in the age of the patients and the reference to partly unilateral and partly bilateral fractures. In future, more comparable studies from selected studies should be analyzed by means of meta-analyses.

Overall, it can be concluded that optimum treatment results can only be achieved through interdisciplinary patient care involving trauma surgery, oral and maxillofacial surgery, and orthodontics.

## 5. Conclusions

All of the studies reported here support functional orthodontic therapy and rehabilitation of mandibular condyle fractures. The treatment outcome, the healing process, the function, and the control of the treatment’s success testify favorably to the treatment of mandibular condyle fracture by activators. Further, the X-rays and the studies show that the remodeling effect is very efficient for the physiological and anatomical restoration of the temporo-mandibular structures. The subjective patient satisfaction investigated in the individual studies was equally satisfactory due to this functional therapy variant. Future studies should investigate subjective patient satisfaction after treatment, comparing interdisciplinary procedures, surgery procedures, and functional orthodontic therapy cases.

## Figures and Tables

**Table 1:** Relevant studies of the systematic review and their summaries.

Author (Year)	Material	Results
Liu et al. [24] (2014)	Follow-up Study, N = 30, Children, Activator, Clinical and X-ray Examination (OPG, CT)	Restitutio ad integrum (N = 20) and Functional remodeling (N = 10), complete (N = 19) and partial (N = 11) X-ray remodeling.
Zhao et al. [23] (2014)	Follow-up Study, N = 48, Children, Activator, Clinical and X-ray Examination (OPG)	Functional capability restored. Bilateral fractures with complete X-ray remodeling, unilateral fractures with incomplete remodeling.
Boffano et al. [22] (2012)	Follow-up study, N = 14, children, IMF + activator + physiotherapy, clinical and X-ray examination (OPG)	Activator therapy for dislocated mandibular condyle fractures in children leads to good functional outcomes. X-ray complete remodeling (N = 13), incomplete remodeling N = 1 without functional correlate.
Wu et al. [21] (2012)	Follow-up study, N = 13, children, IMF + activator + physiotherapy, clinical and X-ray examination	Increase in the maximum mouth opening area and satisfactory results in terms of condyle remodeling and symmetry.
Choi et al. [20] (2005)	Follow-up study, N = 11, children, IMF + activator, clinical and X-ray examination (OPG, CT)	Functional capability restored. Incomplete remodeling (N = 6), complete remodeling (N = 5).
Strobl et al. [19] (1999)	Prospective Study, N = 55, Children, Activator, Clinical and X-ray Examination (OPG)	Functional capability restored. Complete remodeling (N = 47), incomplete remodeling (N = 8) affected the older children aged 7–10 years.
Kahl-Nieke and Fischbach [17] (1998)	Follow-up study, N = 29, children, activator, X-ray examination (CT)	Remodeling potential of the jaw joint depends on age and fracture type: young patients with high remodeling potential, deep luxated older patients with limited remodeling potential.
Wichelhaus et al. [16] (1998)	Prospective study, N = 14, surgical conservative + spring activator, clinical investigation (mouth opening)	Spring activator is suitable for functional restoration of the jaw joint. The cutting-edge difference is increased significantly.
Kahl-Nieke and Fischbach [25] (1995)	Prospective Study, N = 12, Children, Clinical and X-ray Examination (CT)	Functional capability restored. Remodeling depending on the type of fracture: good (N = 3, high non-dislocated fractures), moderate (N = 3), insufficient (N = 9).
Kahl-Nieke, Fischbach and Gerlach [29] (1994)	Retrospective study, N = 7, children, activator, clinical and X-ray examination (CT)	Functional capability restored. Different remodeling processes on fractured condyles can only be evaluated with CT.
Schendel et al. [15] (1991)	Follow-up study, N = 29, children, groups: 1. Activator; 2. IMF + activator, clinical and X-ray examination (OPG, MRI)	Activator therapy only shows better functional and morphological results compared to previously immobilized patients.
Spitzer et al. [30] (1991)	Follow-up study, N = 48, children, activator, clinical and X-ray examination (OPG, CT)	Functional capability restored. Limited remodeling, especially in elderly patients and deep fractures.
Kahl und Gerlach [28] (1990)	Follow-up study, N = 21, children, groups: 1. Activator; 2. IMF + activator, 3. IMF + exercise therapy, Clinical and X-ray examination (OPG, lower jaw hemaxial imaging)	Functional capability restored in all groups, but only in 20% anatomical recovery. Remodeling better in the pure activator group.
Sahm und Witt [18] (1989)	Follow-up study, children, N = 12, children, IMF + activator, X-ray examination (CT).	Favorable remodeling for high, unfavorable remodeling for deep and luxated fractures.
Hirschfelder et al. [12] (1987)	Follow-up study, N = 26, group: 1. Activator; 2. Bignath fixation + activator. Clinical and X-ray examination (CT)	Higher remodeling potential and improved functional recovery of the condylar process in high and medium fractures without significant dislocation. Poor remodeling potential for deep and luxated fractures.
Keutken et al. [14] (1983)	Follow-up study, N = 43, children, activator, clinical and X-ray examination (OPG, Clementschitsch X-ray)	Subjectively good functional result. Objectively significant impairment of function in luxated fractures. Slight impairment of function in dislocated fractures. Significant morphological changes in dislocated and luxated fractures.
Lammers et al. [31] (1983)	Follow-up study, N = 52, children, bignath fixation + activator, clinical and X-ray examination	Functional capability restored. Morphological recovery in 2/3 of the cases.
Holtgrave et al. [27] (1975)	Prospective study, N = 18, children, IMF + activator, clinical and X-ray examination (OPG, lateral tomography)	Functional capability restored. The most common complication is lower jaw deviation to the fractured side. No reconstruction of the luxated fragment. The remodeling potential of the under 10 years old is highest.
Pape et al. [13] (1973)	Retrospective study, N = 61, dislocation fractures, IMF, clinical examination	Functional capability restored (2/3 complaint-free). Common residues were mandible deviation and limitation of lateral movement. Functional approach to luxation fractures justified

**Table 2:** Mandibular condyle fracture and its treatment in children with an average age of 10 years.

Fracture Type	Appliance	Treatment Outcome	Complications during the Follow-Up Examination
Non-dislocated mandibular condyle fracture	Activator therapy (Holtgrave et al. [27] (1975); Keutken et al. [14] (1983); Hirschfelder et al. [12] (1987); Kahl und Gerlach [28] (1990); Kahl-Nieke und Fischbach [25] (1995); Kahl-Nieke et al. [29] (1994); Schendel et al. [15] (1991); Strobl et al. [19] (1999); Choi et al. [20] (2005))	Restitutio ad integrum (Holtgrave et al. [27] (1975); Lammers et al. (1983)); Keutken et al. [14] (1983); Kahl-Nieke und Fischbach [25] (1995); Schendel et al. [15] (1991); Strobl et al. [19] (1999); (Choi et al. [20] (2005)) Resorption and remodeling of the temporomandibular joint head due to the functional influence (Kahl und Gerlach [28] (1990); Kahl-Nieke et al. [29] (1994 Strobl et al. [19] (1999); (Choi et al. [20] (2005)) Functional remodeling (Hirschfelder et al. [12] (1987); Schendel et al. [15] (1991); Kahl-Nieke und Fischbach [25] (1995))	Lower jaw deviation to fractured side (Holtgrave et al. [27] (1975); Liu et al. [24] (2014)) Malocclusion (Choi et al. [20] (2005))
	Activator therapy + physiotherapy (Zhao et al. [23] (2014))	Restitutio ad integrum (Zhao et al. [23] (2014))	
	Occlusal splint therapy (Liu et al. [24] (2014))	Restitutio ad integrum (Liu et al. [24] (2014))	
	IMF (Wu et al. [21] (2012))	Restitutio ad integrum (Holtgrave et al. [27] (1975); Wu et al. [21] (2012)) Functional remodeling (Lammers et al. [31] (1983)) Non-functional remodeling (Keutken et al. [14] (1983); Schendel et al. [15] (1991))	Lower jaw deviation to fractured side (Holtgrave et al. [27] (1975)) Multiple dysfunctions (Keutken et al. [14] (1983))
	IMF + activator therapy (Lammers et al. [31] (1983); Kahl und Gerlach [28] (1990); Schendel et al. [15] (1991))		
	IMF + physiotherapy (Kahl und Gerlach [28] (1990))		
Dislocated mandibular condyle fracture	IMF (Pape et al. [13] (1973); Holtgrave et al. [27] (1975); Kahl und Gerlach [28] (1990); Schendel et al. [15] (1991); Wu et al. [21] (2012)) IMF + physiotherapy (Kahl und Gerlach [28] (1990))	Reconstruction possible (Pape et al. [13] (1973); Wu et al. (2012)) Healing in fractured position (Pape et al. [13] (1973); Wu et al. [21] (2012)) Non-functional remodeling (Schendel et al. [15] (1991))	Lower jaw deviation to fractured side (Pape et al. [13] (1973); Holtgrave et al. [27] (1975); Kahl und Gerlach [28] (1990))
		Dislocated fragment is resorbed and new capitulum is regenerated (Holtgrave et al. [27] (1975))	
	Activator therapy Keutken et al. [14] (1983); (Hirschfelder et al. [12] (1987); Kahl und Gerlach [28] (1990); Kahl-Nieke und Fischbach [25] (1995); Schendel et al. [15] (1991); Spitzer et al. [30] (1991))	Non-functional remodeling (Keutken et al. [14] (1983); Kahl-Nieke und Fischbach [25] (1995))	Morphological changes (Keutken et al. [14] (1983))
		Functional remodeling (Hirschfelder et al. [12] (1987); Schendel et al. [15] (1991); Kahl-Nieke und Fischbach [25] (1995))	
		Shape and positional anomalies in the temporomandibular joint (Spitzer et al. [30] (1991)	
	Occlusal splint therapy (Liu et al. [24] (2014))	Restitutio ad integrum (Liu et al. [24] (2014)) Incomplete remodeling + Functional capability restored (Liu et al. [24] (2014))	
	Activator therapy + physiotherapy (Zhao et al. [23] (2014))	Functional remodeling (Zhao et al. [23] (2014))	
	IMF + activator therapy (Hirschfelder et al. [12] (1987); Wichelhaus et al. [16] (1998); Boffano et al. [22] (2012))	Restitutio ad integrum (Boffano et al. [22] (2012)) Functional remodeling (Hirschfelder et al. [12] (1987); Wichelhaus et al. [16] (1998))	Restricted laterotrusion (Hirschfelder et al. [12] (1987))

## Data Availability

Data are available in the article. Raw data are available from the corresponding author upon reasonable request.
